# Validating the Incorporation of ^13^C and ^15^N in a Shorebird That Consumes an Isotopically Distinct Chemosymbiotic Bivalve

**DOI:** 10.1371/journal.pone.0140221

**Published:** 2015-10-12

**Authors:** Jan A. van Gils, Mohamed Vall Ahmedou Salem

**Affiliations:** 1 NIOZ Royal Netherlands Institute for Sea Research, 1790 AB Den Burg, The Netherlands; 2 EBIOME Ecobiologie Marine et Environnement, Département de Biologie, Université des Sciences, de Technologie et de Médecine, B.P. 880, Nouakchott, Mauritania; 3 Laboratoire de Biologie Appliquée et Pathologie, Département de Biologie, Faculté des Science, B.P. 2121, Tetouan, Morocco; 4 Parc National du Banc d’Arguin, B.P. 5355, Nouakchott, Mauritania; Stockholm University, SWEDEN

## Abstract

The wealth of field studies using stable isotopes to make inferences about animal diets require controlled validation experiments to make proper interpretations. Despite several pleas in the literature for such experiments, validation studies are still lagging behind, notably in consumers dwelling in chemosynthesis-based ecosystems. In this paper we present such a validation experiment for the incorporation of ^13^C and ^15^N in the blood plasma of a medium-sized shorebird, the red knot (*Calidris canutus canutus*), consuming a chemosymbiotic lucinid bivalve (*Loripes lucinalis*). Because this bivalve forms a symbiosis with chemoautotrophic sulphide-oxidizing bacteria living inside its gill, the bivalve is isotopically distinct from ‘normal’ bivalves whose food has a photosynthetic basis. Here we experimentally tested the hypothesis that isotope discrimination and incorporation dynamics are different when consuming such chemosynthesis-based prey. The experiment showed that neither the isotopic discrimination factor, nor isotopic turnover time, differed between birds consuming the chemosymbiotic lucinid and a control group consuming a photosynthesis-based bivalve. This was true for ^13^C as well as for ^15^N. However, in both groups the ^15^N discrimination factor was much higher than expected, which probably had to do with the birds losing body mass over the course of the experiment.

## Introduction

Since its first applications in animal ecology in the 1980s (e.g. [1]), stable-isotope analyses have gained enormous popularity, mostly to identify trophic interactions between species [2], but also to make inferences about global migration patterns [3] or nutrient allocation [4]. Although these studies have generated many insights, progress is sometimes hampered by a proper mechanistic underpinning of these results, as knowledge about isotope incorporation dynamics and discrimination factors may be lacking. The discrimination factor, i.e. the difference in isotope ratio between tissue and diet, is considered *the* most important parameter when it comes to estimating assimilated diets on the basis of stable-isotope analysis [5]. This calls for validation experiments in which consumers are offered food of known isotopic composition and whose tissues are sampled for stable-isotope analysis at regular intervals. In spite of several pleas for such experiments over the past decades [6,7], the number of validation studies is still lagging behind the myriad of field studies applying stable-isotope analysis.

The validation studies that have been done are all performed with organisms living in photosynthesis-based food webs [8–10]. While isotopic field studies of consumers in so-called chemosynthesis-based food webs are also numerous (reviewed by [11,12,13]), to the best of our knowledge controlled validation experiments are lacking, possibly because it is harder to keep such organisms under controlled laboratory conditions [14]. Chemosynthetic ecosystems, which include hydrothermal vents, cold seeps, mud volcanoes and shallow-water coastal sediments, are fuelled by reduced chemical substances such as H_2_S, H_2_, Fe and hydrocarbons such as CH_4_ [15]. By oxidizing these substances, chemoautotrophic bacteria synthesize sugars, which then enter the food web, often through endosymbiosis with invertebrates (including sponges, nematodes, molluscs, and crustaceans). The specificities of anabolic enzymes in chemoautotrophic bacteria cause different isotope discriminations compared to phototrophs [16,17], which is why isotopic signatures in chemosynthetic ecosystems are often unique [18]. Notably invertebrates hosting thiotrophic (sulphur-oxidizing) bacteria show strongly depleted isotopic signatures, both for nitrogen and carbon [19,20].

In this paper we present a validation study of a shorebird (the red knot, *Calidris canutus canutus*) that consumes a chemosymbiotic lucinid bivalve (*Loripes lucinalis*; *Loripes* from now on). Red knots are long-distance migrants that, in the case of the *canutus* subspecies, overwinter in the subtropical intertidal ecosystem of Banc d’Arguin (Mauritania, West-Africa) [21]. Here, *Loripes* is by and large the most abundant prey species [22–24], and makes up a significant proportion of the red knots’ diet [22,25,26], in spite of mildly toxic effects during digestion [27]. These toxic effects are due to sulphide-oxidation taking place in the bivalve’s gill by endosymbiotic bacteria. Because of a strong reliance on this sulphur-based metabolism [28], *Loripes* shows strongly depleted ^15^N/^14^N and ^13^C/^12^C ratios [28–31]. As stable-isotope ratios of the food affect isotope discrimination in the consumer [32], we hypothesize that *Loripes*-consuming red knots show distinct incorporation dynamics and isotopic discrimination. In order to test this hypothesis, a group of *Loripes*-consuming red knots was contrasted with a control group consuming a venerid bivalve, *Dosinia isocardia*, which has a ‘normal’ photosynthesis-based isotopic signature [33] (*Dosinia* from now on; however note a recent change in this species genus name to *Pelecyora* [34]).

## Materials and Methods

In the evening of 20 January 2012, 61 red knots were caught with mistnets at the high-tide roost at Abelgh Eiznaya, Banc d’Arguin, Mauritania [35], from which we randomly selected six adult individuals to participate in this validation experiment (some of the remaining 55 birds were kept for other experiments [36,37], the rest was released immediately after ringing). Birds were randomly assigned to two groups of three birds each and were housed in small pens (1.5 × 1.0 × 0.5 m) at the Iwik biological station. During the first four days we allowed the birds to get habituated to captivity while they were fed a mixture of *Loripes*, *Dosinia* and the flesh of large *Senilia senilis*. On the fifth day of captivity the experiment started (i.e. experimental day 0 in the analyses and graphs below). From then onwards, one group of birds was offered *Loripes* only, while the other group of birds was offered *Dosinia* only. This food was offered *ad libitum* in small trays, which were refilled every morning, for a period of 19 days, until the end of the experiment. Based on an earlier validation study in red knots [10], we anticipated that such a relatively short period would be sufficient for the stable-isotope ratios to reach equilibrium in the blood plasma, but not in the red blood cells. The birds always had access to freshwater. Each day, *Loripes* was collected in the seagrass beds of Abelgh Eiznaya (2 km NW from the station), while *Dosinia* was gathered from the nearest sandy beach (250 m E from the station; for both prey species using a sieve with a 2-mm mesh size). These prey were kept in a refrigerator until they were offered to the birds (same or next day). The birds’ body mass was measured daily, in order to monitor health status and to try to keep them at a stable body mass throughout the experiment (as changes in body mass may interfere with isotopic discrimination factors [38–40]). At experimental days 0, 5, 10, 16 and 19 we took a small blood sample from each bird for the purpose of stable isotope analysis. To this end, we punctured the wing vein and collected a small volume of blood (60–120 μL) into 75-μL heparinized capillaries. Next, capillaries were emptied into 1.5-mL microcentrifuge tubes. After all six birds were sampled these tubes were centrifuged (12 min at 6900 *g*) to separate plasma from red blood cells. Plasma and cell samples were kept frozen until stable isotope analysis at NIOZ, where they were freeze-dried to constant mass [41], where after 0.4–0.8 mg of freeze-dried material (determined with a Sartorius XM1000P microbalance) was deposited into 5 × 9 mm tin capsules. These small subsamples were then analysed in a Thermo Scientific FLASH 2000 organic element analyser coupled to a Delta V isotope ratio mass spectrometer. A laboratory acetanilide standard with *δ* ^13^C and *δ* ^15^N values calibrated against NBS-22 oil and IAEA-N1, respectively, was used for calibration. The average repeatability of *δ* ^13^C and *δ* ^15^N determination was 0.04 ‰ (n = 22) and 0.21 ‰ (n = 22), respectively, based on repeated analysis of the acetanilide standard over time.

Following standard practice, we expressed *δ* ^13^C and *δ* ^15^N values in units of per mil (‰) difference from the *δ* ^13^C_VPDB_ and *δ* ^15^N_Air_ reference values, respectively [42,43]. To statistically model the dynamics of isotopic incorporation in the tissue over time, we used the widely-used one-compartment exponential decay function [10,44]:

$$\delta (t)=\delta (\infty )+(\delta (0)-\delta (\infty ))\times e^{-\lambda t}$$ (1)

For either carbon or nitrogen, in either plasma or cells, *δ*(*t*) is the stable isotope ratio at time *t* (being either 0, 5, 10, 16 or 19 days), *δ*(0) is the isotope ratio at *t* = 0, *δ*(∞) is the asymptote at which the isotopic value of the tissue is in equilibrium with the new diet, and *λ* is the instantaneous incorporation rate of the element in the tissue [45]. These functions were fitted in nonlinear mixed-effect models, using the *nlme* package [46] in *R* [47], in which estimates for *δ*(0) were included as random between-individual effects. Discrimination factors *Δ* were calculated as *Δ* = *δ*(∞)– *δ*_diet_, in which estimates for *δ*_diet_ were taken from Catry *et al*. [48], who collected *Dosinia* and *Loripes* in our study area in two subsequent winters (2012/2013 and 2013/2014) and determined the following entire soft tissue stable-isotope ratios (± SE): *δ* ^13^C_*Dosinia*_ = –15.88 ‰ (± 0.58 ‰), *δ* ^15^N_*Dosinia*_ = 6.48 ‰ (± 0.31 ‰), *δ* ^13^C_*Loripes*_ = –24.50 ‰ (± 0.29 ‰), and *δ* ^15^N_*Loripes*_ = 0.53 ‰ (± 0.35 ‰). Although isotopic signatures may vary seasonally, interannual variations are negligible [28].

### Ethics statement

The experiment was performed under full permission by the authorities of the Parc National du Banc d’Arguin (PNBA). No animal experimentation ethics guidelines exist in Mauritania. However, the experiment was carried out in strict accordance with Dutch animal experimentation guidelines. The NIOZ Royal Netherlands Institute for Sea Research has been licensed by the Dutch Ministry of Health to perform animal experiments under license number 80200. This license involves capture and handling of animals, and performing experiments, which nonetheless should be individually approved by the Animal Experimentation Committee (DEC) of the Royal Netherlands Academy of Arts and Sciences (KNAW). The DEC does not provide permits for experiments in foreign countries, but provided approval for equivalent experiments in the Netherlands under permit number NIOZ 10.05, involving the capture of red knots, performing experiments consisting of prolonged diets of natural food types (i.e. foods that regularly occur in the diet of wild red knots), and includes permission to release healthy animals in the wild after the experiment. All possible efforts were made to minimize physical and mental impact on the experimental animals. After the experiment ended, the birds were given *ad libitum* quantities of the flesh of large *Senilia senilis* for a couple of days, such that they regained body mass before release in the wild.

## Results

**Fig 1:**
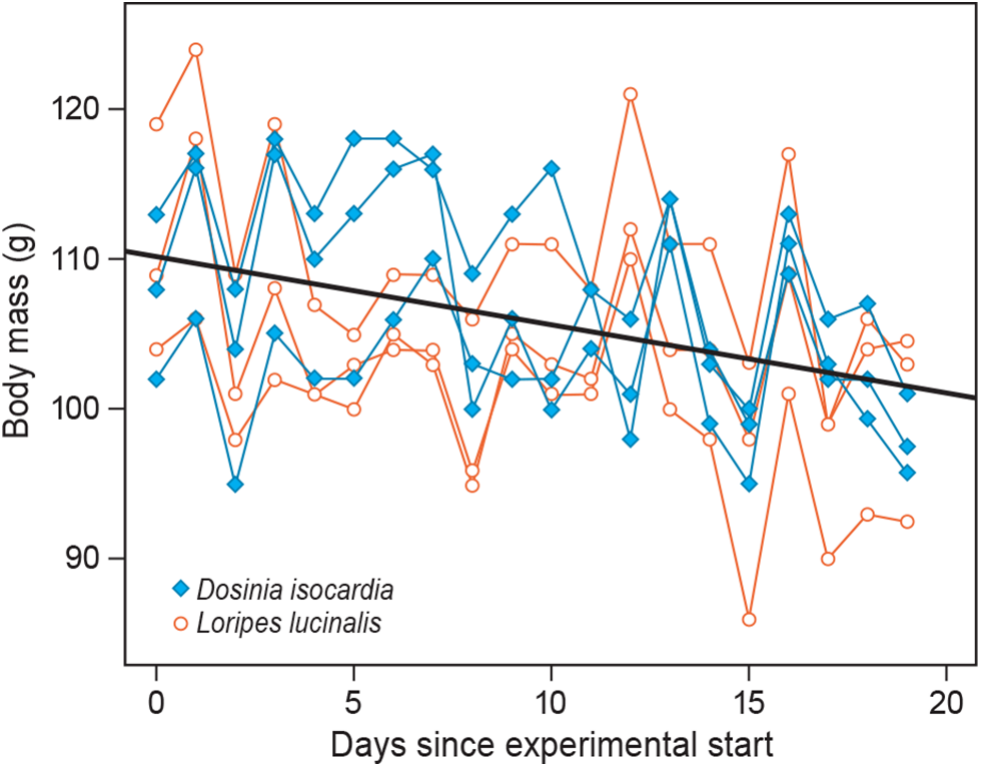
Daily body mass throughout the experiment. Individual data are connected and diet is given in legend. Thick straight line represents mixed-effect model fit.

Over the course of the experiment the birds lost body mass (Fig 1) at an average rate of 0.5 g/day (*t* = -5.34, df = 113, *P* < 0.0001) with no differences between groups (*t* = 0.66, df = 4, *P* = 0.54; mixed-effect model with a random intercept for each bird).

**Fig 2:**
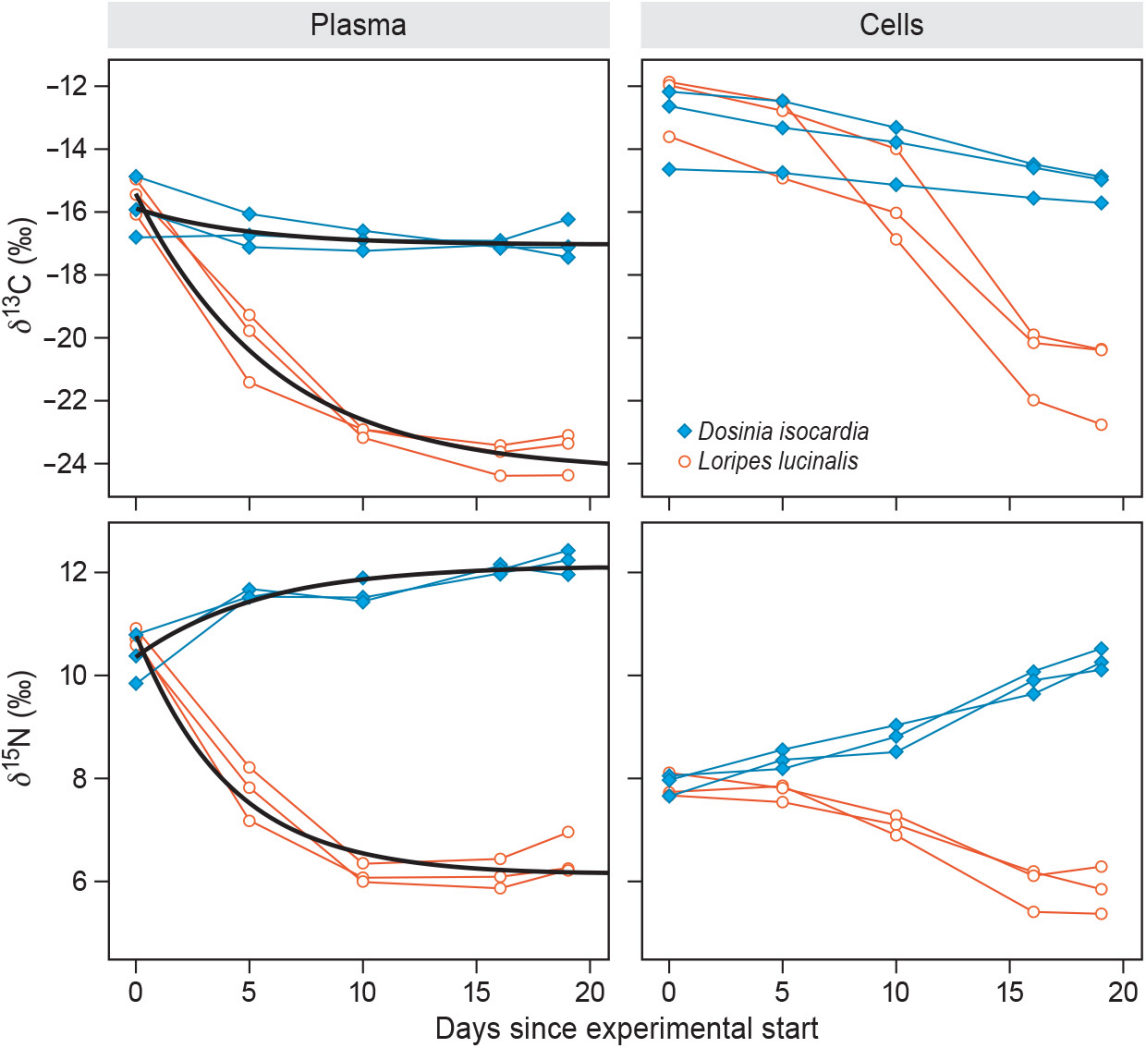
Isotope ratios *δ* ^13^C (upper panels) and *δ* ^15^N (lower panels) throughout the experiment in blood plasma (left panels) and red blood cells (right panels). Individual measurements are connected, diets are given in legend (upper right panel), and thicker lines denote nonlinear mixed-effect model fits (plasma only; model fits failed to converge for red blood cells).

The exponential decay function (Eq 1) was fitted to the plasma data (Table 1), but failed to convergence in the case of the red blood cell data (Fig 2). Zooming in on the results for plasma, estimates for *δ*(0) did, as expected, not differ between groups, either for *δ* ^13^C (mean ± SE = –15.66 ± 0.34 ‰), or for *δ* ^15^N (10.57 ± 0.16 ‰). Also the estimates for *λ* did not vary between groups and were statistically indistinguisable for *δ* ^13^C and *δ* ^15^N, averaging out at 0.20 day^-1^ (SE = 0.03 day^-1^). As expected, estimates for *δ*(∞) *did* differ between groups, both for *δ* ^13^C and for *δ* ^15^N (see Table 1 for estimates).

**Table 1:** Parameter estimates for the one-compartment exponential decay function fitted to the plasma stable isotope ratios (± SE). *δ*(0) is the isotope ratio at the start of the experiment, *δ*(∞) is the asymptote of the isotope ratio, and *λ* is the instantaneous incorporation rate of the element.

	*δ* ^13^C			*δ* ^15^N		
	*δ*(0) [‰]	*δ*(∞) [‰]	*λ* (day^-1^)	*δ*(0) [‰]	*δ*(∞) [‰]	*λ* (day^-1^)
*Dosinia isocardia*	–15.88 ± 0.52	–17.03 ± 0.16	0.21 ± 0.10	10.36 ± 0.21	12.14 ± 0.15	0.19 ± 0.06
*Loripes lucinalis*	–15.43 ± 0.44	–24.28 ± 0.45	0.17 ± 0.03	10.77 ± 0.24	6.12 ± 0.20	0.24 ± 0.05

**Fig 3:**
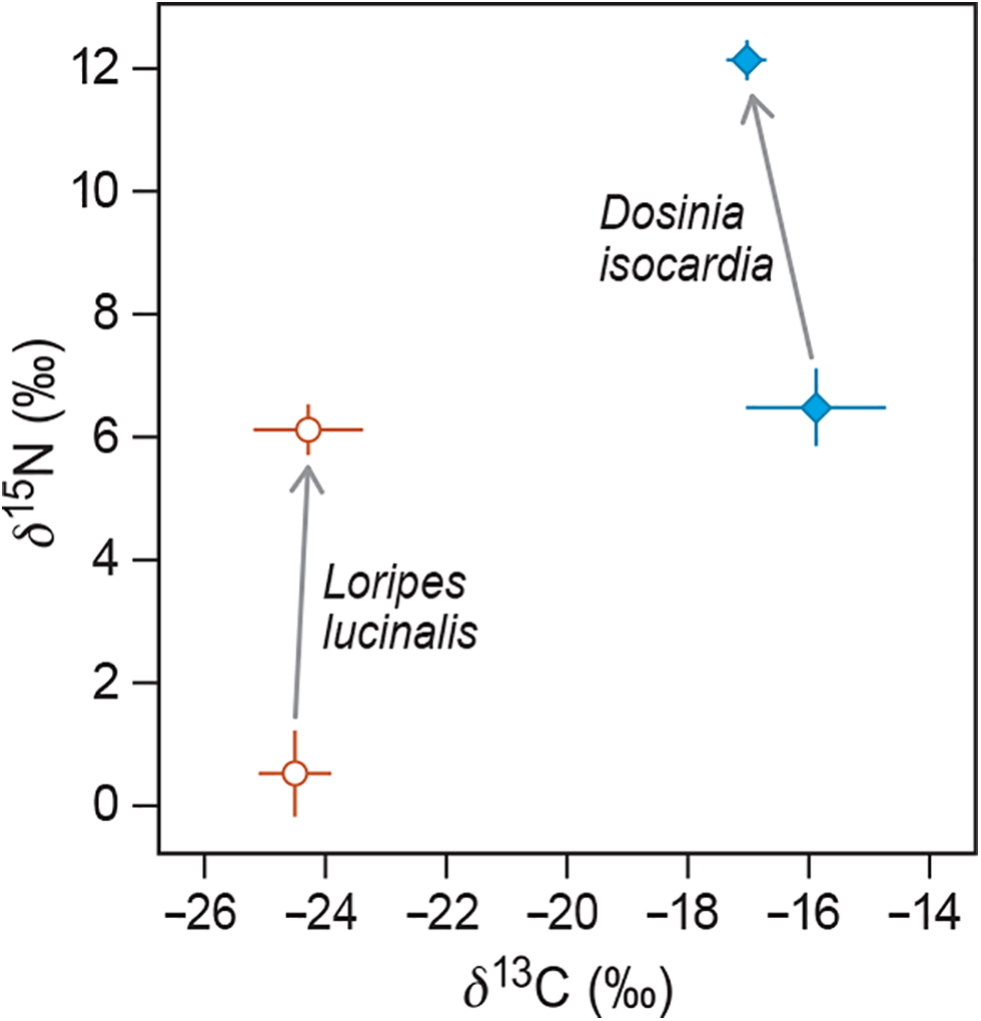
Discrimination of *δ* ^13^C and *δ* ^15^N from food (at arrow base) to plasma (at arrow head), plotted for the two diet groups separately. Bars represent 95% confidence intervals.

These estimates of *δ*(∞) in plasma enabled us to calculate diet-plasma discrimination factors *Δ* (Fig 3). For *δ* ^13^C this yielded *Δ δ* ^13^C _*Dosinia*_ (± SE) = –1.15 ‰ (± 0.60 ‰) and *Δ δ* ^13^C _*Loripes*_ = +0.22 ‰ (± 0.54 ‰). For *δ* ^15^N this yielded *Δ δ* ^15^N _*Dosinia*_ = +5.66 ‰ (± 0.35 ‰) and *Δ δ* ^15^N _*Loripes*_ = +5.59 ‰ (± 0.40 ‰).

## Discussion

In the literature, average (± SD) discrimination factors are +0.4 ‰ (± 1.3 ‰) for *δ* ^13^C and +3.4 ‰ (± 1.0 ‰) for *δ* ^15^N [49–52]. For *δ* ^13^C our discrimination factor estimates are not too far off from these widely-used figures (or even statistically indistinguishable in the case of *Loripes*). However, for *δ* ^15^N we find much higher discrimination factors than normally observed (5.66 and 5.59 ‰ for *Dosinia* and *Loripes*, respectively). Note that relatively high values have also been observed in a closely related shorebird species (the dunlin, *Calidris alpina*) [53], but not always [54]. The fact that in our study *Δ δ* ^15^N was not only high in the *Loripes* group, but also in the *Dosinia* group, rejects the hypothesis that the unique chemoautotrophic signature of *Loripes* isotopes has an effect on the discrimination factor. Instead, these high values are very likely due to the fact that our birds were losing body mass over the course of the experiment (Fig 1; at a similar rate in both groups), a result which presumably had to do with our inability to collect enough food for six birds on a daily basis (trays were often emptied overnight). It is well established that *δ* ^15^N discrimination factors are higher in animals losing lean mass during nutritional stress [38–40]. This is because nitrogeneous waste products (such as uric acid) have a low *δ* ^15^N relative to body nitrogen ('catabolic model' in [38]), and because starving animals show increased recycling of nitrogen leading to ‘discrimination on top of discrimination’ during protein synthesis ('anabolic model' in [38]). Body mass in our birds declined from approx. 110 g to approx. 100 g, which is the range in which red knots deplete their own protein stores [39,55–57]. Alternative hypotheses explaining high values for *Δ δ* ^15^N, the protein-quality hypothesis and the protein-quantity hypothesis [5], predict poor-quality protein and a high protein content of the food, respectively. However, our results reject both hypotheses. Values for *Δ δ* ^15^N > 5 ‰ have only been found in consumers of poor-quality plant matter [5], which rejects the protein-quality hypothesis. The protein-quantity hypothesis is rejected because, although shellfish *do* contain high amounts of protein (75% in [39]), the birds did not obtain enough of it as indicated by their body mass loss. Moreover, up to now, protein-quantity effects on *Δ δ* ^15^N have never exceeded levels beyond 1 ‰ [5,58,59].

The observed instantaneous incorporation rates *λ* were found to be independent of isotope and diet, which thereby rejects the hypothesis that incorporation dynamics are affected by the chemoautotrophic nature of the food. Average *λ* was 0.20 day^-1^, which is equivalent to a residence time *τ* of 5.0 days (1/*λ*), and a half-life *t*_½_ of 3.4 days (ln(2)/*λ*) [6]. Among other tissues, plasma is known to have relatively short turnover times [60]. The longer turnover time normally observed in red blood cells (e.g. τ = 21.7 days in reference [10]), is most likely the reason that our nonlinear mixed-effect models failed to converge on the red blood cell data–with 19 days our experiment simply lasted not long enough for the red blood cells to achieve an isotopic equilibrium state.

The observed 3.4 days plasma half-life is somewhat shorter than an earlier estimate in red knots of 4.8 days [10], but is similar to the allometrically predicted half-life of 3.2 days for avian plasma by Vander Zanden *et al*. [61] and falls in between the allometric predictions derived separately for *δ* ^13^C (3.8 days) and *δ* ^15^N (2.0 days) by Thomas & Crowther [62] (using the observed average body mass of 105 g). This allometric congruency is promising and may become helpful when making inferences about the *timing* of diet shifts on the basis of blood tissue stable-isotope ratios. Such diet shifts are often indicative of a migratory movement in migrants that travel between isotopically distinct habitats, such as marine and terrestrial habitats in the case of the red knot [10,41]. With Banc d’Arguin being a chemosynthesis-based ecosystem, and thus being isotopically distinct from photosynthesis-based stopovers along the red knot’s flyway, we may even be able to make inferences about departure/arrival timing from/to Banc d’Arguin in future studies. This would then be possible at times when photosynthesis-based bivalves such as *Dosinia* are scarce, as then red knots rely heavily on *Loripes* [22].

## References

[1] DeNiro MJ, Epstein S (1981) Influence of diet on the distribution of nitrogen isotopes in animals. Geochimica et Cosmochimica Acta 45: 341–351.

[2] Layman CA, Araujo MS, Boucek R, Hammerschlag-Peyer CM, Harrison E, Jud ZR, et al (2012) Applying stable isotopes to examine food-web structure: an overview of analytical tools. Biological Reviews 87: 545–562. 10.1111/j.1469-185X.2011.00208.x 22051097

[3] Rubenstein DR, Hobson KA (2004) From birds to butterflies: animal movement patterns and stable isotopes. Trends in Ecology & Evolution 19: 256–263. 16701265 10.1016/j.tree.2004.03.017

[4] Klaassen M, Lindström Å, Meltofte H, Piersma T (2001) Arctic waders are not capital breeders. Nature 413: 794. 10.1038/35101654 11677593

[5] Florin ST, Felicetti LA, Robbins CT (2011) The biological basis for understanding and predicting dietary-induced variation in nitrogen and sulphur isotope ratio discrimination. Functional Ecology 25: 519–526.

[6] Martínez del Rio C, Wolf N, Carleton SA, Gannes LZ (2009) Isotopic ecology ten years after a call for more laboratory experiments. Biological Reviews 84: 91–111. 10.1111/j.1469-185X.2008.00064.x 19046398

[7] Gannes LZ, Obrien DM, del Rio CM (1997) Stable isotopes in animal ecology: assumptions, caveats, and a call for more laboratory experiments. Ecology 78: 1271–1276.

[8] Caut S, Laran S, Garcia-Hartmann E, Das K (2011) Stable isotopes of captive cetaceans (killer whales and bottlenose dolphins). Journal of Experimental Biology 214: 538–545. 10.1242/jeb.045104 21270301

[9] Caut S (2013) Isotope incorporation in broad-snouted caimans (crocodilians). Biology Open 2: 629–634. 10.1242/bio.20134945 23789113 PMC3683165

[10] Klaassen M, Piersma T, Korthals H, Dekinga A, Dietz MW (2010) Single-point isotope measurements in blood cells and plasma to estimate the time since diet switches. Functional Ecology 24: 796–804.

[11] Kennicutt MC, Burke RA, Macdonald IR, Brooks JM, Denoux GJ, Macko SA (1992) Stable isotope partitioning in seep and vent organisms–chemical and ecological significance. Chemical Geology 101: 293–310.

[12] Van Dover CL (2007) Stable isotope studies in marine chemoautotrophically based ecosystems: an update In: Michener R, Lajtha K, editors. Stable Isotopes in Ecology and Environmental Science. Oxford, UK: Blackwell Publishing Ltd. pp. 202–237.

[13] Conway N, Kennicutt MC, Van Dover CL (1994) Stable isotopes, microbial symbioses and microbial physiology In: Lajtha K, Michener R, editors. Stable Isotopes in Ecology. Oxford, UK: Blackwell Scientific Publications pp. 158–188.

[14] Van Dover CL, Lutz RA (2004) Experimental ecology at deep-sea hydrothermal vents: a perspective. Journal of Experimental Marine Biology and Ecology 300: 273–307.

[15] Dubilier N, Bergin C, Lott C (2008) Symbiotic diversity in marine animals: the art of harnessing chemosynthesis. Nature Reviews Microbiology 6: 725–740. 10.1038/nrmicro1992 18794911

[16] Hoch MP, Fogel ML, Kirchman DL (1992) Isotope fractionation associated with ammonium uptake by a marine bacterium. Limnology and Oceanography 37: 1447–1459.

[17] Ruby EG, Jannasch HW, Deuser WG (1987) Fractionation of stable carbon isotopes during chemoautotrophic growth of sulfur-oxidizing bacteria. Applied and Environmental Microbiology 53: 1940–1943. 16347420 10.1128/aem.53.8.1940-1943.1987 PMC204029

[18] Mae A, Yamanaka T, Shimoyama S (2007) Stable isotope evidence for identification of chemosynthesis-based fossil bivalves associated with cold-seepages. Palaeogeography, Palaeoclimatology, Palaeoecology 245: 411–420.

[19] Dreier A, Loh W, Blumenberg M, Thiel V, Hause-Reitner D, Hoppert M (2014) The isotopic biosignatures of photo- vs. thiotrophic bivalves: are they preserved in fossil shells? Geobiology 12: 406–423. 10.1111/gbi.12093 25039581

[20] Dreier A, Stannek L, Blumenberg M, Taviani M, Sigovini M, Wrede C, et al (2012) The fingerprint of chemosymbiosis: origin and preservation of isotopic biosignatures in the nonseep bivalve *Loripes lacteus* compared with *Venerupis aurea* . FEMS Microbiology Ecology 81: 480–493. 10.1111/j.1574-6941.2012.01374.x 22458451

[21] Piersma T (2007) Using the power of comparison to explain habitat use and migration strategies of shorebirds worldwide. Journal of Ornithology 148: S45–S59.

[22] van Gils JA, van der Geest M, Leyrer J, Oudman T, Lok T, Onrust J, et al (2013) Toxin constraint explains diet choice, survival and population dynamics in a molluscivore shorebird. Proceedings of the Royal Society B: Biological Sciences 280: 20130861 10.1098/rspb.2013.0861 23740782 PMC3774237

[23] Honkoop PJC, Berghuis EM, Holthuijsen S, Lavaleye MSS, Piersma T (2008) Molluscan assemblages of seagrass-covered and bare intertidal flats on the Banc d’Arguin, Mauritania, in relation to characteristics of sediment and organic matter. Journal of Sea Research 60: 255–263.

[24] Ahmedou Salem MV, van der Geest M, Piersma T, Saoud Y, van Gils JA (2014) Seasonal changes in mollusc abundance in a tropical intertidal ecosystem, Banc d’Arguin (Mauritania): testing the ‘depletion by shorebirds’ hypothesis. Estuarine, Coastal and Shelf Science 136: 26–34.

[25] Onrust J, de Fouw J, Oudman T, van der Geest M, Piersma T, van Gils JA (2013) Red knot diet reconstruction revisited: context dependence revealed by experiments at Banc d’Arguin, Mauritania. Bird Study 60: 298–307.

[26] van Gils JA, van der Geest M, Jansen EJ, Govers LL, de Fouw J, Piersma T (2012) Trophic cascade induced by molluscivore predator alters pore-water biogeochemistry via competitive release of prey. Ecology 93: 1143–1152. 22764500 10.1890/11-1282.1

[27] Oudman T, Onrust J, de Fouw J, Spaans B, Piersma T, van Gils JA (2014) Digestive capacity and toxicity cause mixed diets in red knots that maximize energy intake rate. American Naturalist 183: 650–659. 10.1086/675759 24739197

[28] van der Geest M, Sall AA, Ely SO, Nauta RW, van Gils JA, Piersma T (2014) Nutritional and reproductive strategies in a chemosymbiotic bivalve living in a tropical intertidal seagrass bed. Marine Ecology—Progress Series 501: 113–126.

[29] Johnson M, Diouris M, Le Pennec M (1994) Endosymbiotic bacterial contribution in the carbon nutrition of *Loripes lucinalis* (Mollusca: Bivalvia). Symbiosis 17: 1–13.

[30] Le Pennec M, Herry A, Johnson M, Beninger P (1995) Nutrition-gametogenesis relationship in the endosymbiont host-bivalve *Loripes lucinalis* (Lucinidae) from reducing coastal habitats. In: Eleftheriou A, Ansell AD, Smith CJ, editors. Biology and Ecology of Shallow Coastal Waters—Proceedings of the 28th European Marine Biological Symposium, Crete, Greece, 23–28 September 1993. Copenhagen: Olsen & Olsen. pp. 139–142.

[31] Rossi F, Colao E, Martinez MJ, Klein JC, Carcaillet F, Callier MD, et al (2013) Spatial distribution and nutritional requirements of the endosymbiont-bearing bivalve *Loripes lacteus* (sensu Poli, 1791) in a Mediterranean *Nanozostera noltii* (Hornemann) meadow. Journal of Experimental Marine Biology and Ecology 440: 108–115.

[32] Caut S, Angulo E, Courchamp F (2009) Variation in discrimination factors (*Δ* ^15^N and *Δ* ^13^C): the effect of diet isotopic values and applications for diet reconstruction. Journal of Applied Ecology 46: 443–453.

[33] van der Geest M (2013) Multi-trophic interactions within the seagrass beds of Banc d’Arguin, Mauritania: PhD thesis, University of Groningen, The Netherlands.

[34] Huber M (2015) *Pelecyora isocardia* (Dunker, 1845). In: Bouchet P, Gofas S, Rosenberg G, Bank RA, Bieler R, editors. MolluscaBase: World Register of Marine Species. Available: http://www.marinespecies.org/aphia.php?p=taxdetails&id=507810.

[35] Leyrer J, Lok T, Brugge M, Dekinga A, Spaans B, van Gils JA, et al (2012) Small-scale demographic structure suggests preemptive behavior in a flocking shorebird. Behavioral Ecology 23: 1226–1233.

[36] Oudman T, Hin V, Dekinga A, van Gils JA (2015) The effect of digestive capacity on the intake rate of toxic and non-toxic prey in an ecological context. PLoS ONE 10: e0136144 10.1371/journal.pone.0136144 26287951 PMC4543589

[37] de Fouw J, Kok EMA, Penning E, Piersma T, van Gils JA (n.d.) Multiple dimensions of habitat selection: how the combined effects of food density and detectability drive forager distributions. In press.

[38] Lee TN, Buck CL, Barnes BM, O'Brien DM (2012) A test of alternative models for increased tissue nitrogen isotope ratios during fasting in hibernating arctic ground squirrels. Journal of Experimental Biology 215: 3354–3361. 10.1242/jeb.068528 22735347

[39] Dietz MW, Piersma T, Dekinga A, Korthals H, Klaassen M (2013) Unusual patterns in ^15^N blood values after a diet switch in red-knot shorebirds. Isotopes in Environmental and Health Studies 49: 283–292. 10.1080/10256016.2013.776045 23656233

[40] Hobson KA, Alisauskas RT, Clark RG (1993) Stable-nitrogen isotope enrichment in avian tissues due to fasting and nutritional stress: implications for isotopic analyses of diet. Condor 95: 388–394.

[41] Dietz MW, Spaans B, Dekinga A, Klaassen M, Korthals H, van Leeuwen C, et al (2010) Do red knots (*Calidris canutus islandica*) routinely skip Iceland during southward migration? Condor 112: 48–55.

[42] Coplen TB (1996) More uncertainty than necessary. Paleoceanography 11: 369–370.

[43] Bond AL, Hobson KA (2012) Reporting stable-isotope ratios in ecology: recommended terminology, guidelines and best practices. Waterbirds 35: 324–331.

[44] Martínez del Rio C, Wolf BO (2005) Mass balance models for animal isotopic ecology In: Starck MA, Wang T, editors. Physiological and Ecological Adaptations to Feeding in Vertebrates. Enfield, New Hampshire: Science Publishers pp. 141–172.

[45] Martínez del Rio C, Carleton SA (2012) How fast and how faithful: the dynamics of isotopic incorporation into animal tissues. Journal of Mammalogy 93: 353–359.

[46] Pinheiro JC, Bates D, DebRoy S, Sarkar D, R Core Team (2015) nlme: linear and nonlinear mixed effect models. R package version 31–120.

[47] R Core Team (2013) R: A language and environment for statistical computing Vienna, Austria: R Foundation for Statistical Computing.

[48] Catry T, Lourenço PM, Lopes RJ, Carneiro C, Alves JA, Costa J, et al (2015) Structure and functioning of intertidal food webs along an avian flyway: a comparative approach using stable isotopes. Functional Ecology: Early View, 10.1111/1365-2435.12506

[49] Post DM (2002) Using stable isotopes to estimate trophic position: models, methods, and assumptions. Ecology 83: 703–718.

[50] McCutchan JH, Lewis WM, Kendall C, McGrath CC (2003) Variation in trophic shift for stable isotope ratios of carbon, nitrogen, and sulfur. Oikos 102: 378–390.

[51] Vander Zanden MJ, Rasmussen JB (2001) Variation in *δ* ^15^N and *δ* ^13^C trophic fractionation: implications for aquatic food web studies. Limnology and Oceanography 46: 2061–2066.

[52] Vanderklift MA, Ponsard S (2003) Sources of variation in consumer-diet *δ* ^15^N enrichment: a meta-analysis. Oecologia 136: 169–182. 12802678 10.1007/s00442-003-1270-z

[53] Evans Ogden LJ, Hobson KA, Lank DB, Martínez del Rio C (2004) Blood isotopic (*δ* ^13^C and *δ* ^15^N) turnover and diet-tissue fractionation factors in captive dunlin (*Calidris alpina pacifica*). Auk 121: 170–177.

[54] Lourenço PM, Granadeiro JP, Guilherme JL, Catry T (2015) Turnover rates of stable isotopes in avian blood and toenails: implications for dietary and migration studies. Journal of Experimental Marine Biology and Ecology 472: 89–96.

[55] Piersma T, Poot M (1993) Where waders may parallel penguins: spontaneous increase in locomotor activity triggered by fat depletion in a voluntarily fasting knot. Ardea 81: 1–8.

[56] Piersma T, Bruinzeel L, Drent R, Kersten M, van der Meer J, Wiersma P (1996) Variability in basal metabolic rate of a long-distance migrant shorebird (red knot *Calidris canutus*) reflects shifts in organ sizes. Physiological Zoology 69: 191–217.

[57] Klaassen M, Kersten M, Ens BJ (1990) Energetic requirements for maintenance and premigratory body mass gain of waders wintering in Africa. Ardea 78: 209–220.

[58] Pearson S, Levey D, Greenberg C, Martínez del Rio C (2003) Effects of elemental composition on the incorporation of dietary nitrogen and carbon isotopic signatures in an omnivorous songbird. Oecologia 135: 516–523. 16228250 10.1007/s00442-003-1221-8

[59] Focken U (2001) Stable isotopes in animal ecology: the effect of ration size on the trophic shift of C and N isotopes between feed and carcass. Isotopes in Environmental and Health Studies 37: 199–211. 11924851 10.1080/10256010108033296

[60] Boecklen WJ, Yarnes CT, Cook BA, James AC (2011) On the use of stable isotopes in trophic ecology. Annual Review of Ecology, Evolution, and Systematics 42: 411–440.

[61] Vander Zanden MJ, Clayton MK, Moody EK, Solomon CT, Weidel BC (2015) Stable isotope turnover and half-life in animal tissues: a literature synthesis. PLoS ONE 10: e0116182 10.1371/journal.pone.0116182 25635686 PMC4321325

[62] Thomas SM, Crowther TW (2015) Predicting rates of isotopic turnover across the animal kingdom: a synthesis of existing data. Journal of Animal Ecology 84: 861–870. 10.1111/1365-2656.12326 25482029

